# Profile of audiometric thresholds and tympanometric curve of elderly patients

**DOI:** 10.1590/S1808-86942010000500022

**Published:** 2015-10-22

**Authors:** Tatiana Marques Guerra, Lucimar Pires Estevanovic, Marcela de Ávila Meira Cavalcante, Rafaela Carolina Lopez Silva, Izabel Cristina Campolina Miranda, Victor Gandra Quintas

**Affiliations:** 1 Speech and Hearing Therapist; Audiologist - Pontfícia Universidade Católica de Minas Gerais; 2 Speech and Hearing Therapist; Audiologist - Pontfícia Universidade Católica de Minas Gerais; 3 Speech and Hearing Therapy Student - Pontfícia Universidade Católica de Minas Gerais; 4 MSc in Speech and Hearing Therapy - Pontfícia Universidade Católica de São Paulo; Assistant Professor of Speech and Hearing Therapy - Pontfícia Universidade Católica de Minas Gerais; 5 PhD in Linguistics - Federal University of Minas Gerais; Full Professor of Speech and Hearing Therapy - Pontfícia Universidade Católica de Minas Gerais; 6 Speech and Hearing Therapist; MSc in Human Communication Disorders - Federal University of Santa Maria - RS. Pontfícia Universidade Católica de Minas Gerais

**Keywords:** hearing loss, aged, speech, sensorineural, hearing.

## Abstract

**Abstract:**

Aim: to analyze the audiological profile of elderly patients seen in a clinic from an audiology school clinic in the city of Belo Horizonte.

**Methods:**

we studied all the charts from the patients who underwent audiologic assessment from April of 2004 and August of 2007 in an audiology clinic in the city of Belo Horizonte.

**Results:**

We studied the 313 audiological tests from patients 60 years of age or over. The results from the audiological evaluations as to the type of hearing loss were: auditory thresholds within normal standards - 22.28%; sensorineural hearing loss - 60.62%; mixed hearing loss - 14.70%, conductive hearing loss - 2.40%. The level varied between normal and profound. As to the tympanometry, 83.22% had the type A curve, and the other types of curves obtained made up a total of 16.3%. The percentage of individuals who did not undergo the test was 0.48%. 1.76% of the patients who had unilateral hearing loss and 98.24% had bilateral hearing loss.

**Conclusions:**

we found a greater prevalence of sensorineural hearing loss, and the degree of the loss varied from mild to profound, with a prevalence of the moderate degree.

## INTRODUCTION

Hearing loss is the partial or total hearing disability, which can be associated with birth, acquired diseases, use of ototoxic drugs or aging. This change can affect any age range. Children with hearing loss can have development difficulties, while elderly patients have natural hearing loss with aging, called presbycusis^1^, ^2^.

Quality of life improvement, improvement in health conditions and chronic and infectious diseases control made people live longer. However, the effects of aging on the sensorial capacities did not change, causing diseases stemming from this aging, which is the case of the hearing loss which affects the elderly^2^.

Hearing loss is one of the sensorial deficits which has the most impact on the lives of people; because it impairs the person's capacity to effectively engage in communication. Thus, there is a reduction on speech intelligibility, impairing verbal communication, interfering in receiving information, forming and expressing one's ideas. Moreover, there is patient isolation, reduced socialization and intolerance to moderate to high-intensity sounds^1^, ^2^, ^3^.

Presbycusis is the natural aging of the human ear, in other words, a hearing disorder associated to cochlea degeneration, which affects mainly the base of it, impairing hearing perception in the high frequencies as age increases^1^, ^2^, ^3^, ^4^.

The main symptoms the elderly have associated with presbycusis are: difficulties in participating in talks or speaking on the phone; understanding words; locating a sound source; hearing alarms, the telephone, doorbell, approaching vehicles; and there is the need to raise the volumes of TV or radio^1^, ^2^,^5^.

Presbycusis is defined as sensorineural hearing loss, which varies between mild to profound in the low as well as the high frequencies, having a gradual and progressive onset, symmetrical, descending and bilateral for high frequency sounds (3 to 8KHz), often times followed by difficulties in speech recognition^6^, ^7^, ^8^, ^9^, ^10^.

According to what has been previously reported, presbycusis has a downwards characteristic, being so described by the classification criterion of the hearing loss level of an American study^11^. However; in the present study, we will approach the classification criterion of a Brazilian study^10^.

The elderly's audiologic evaluation must involve subjective and objective exams in order to define his/ her audiologic thresholds. Thus, some factors must be observed, such as understanding and the execution of the orders given to the patient in order to obtain an accurate result on the functional use of hearing and understanding. There must be certainty on the orders given to the patient and to check whether or not they are being understood, because the linguistic implications are directly related to the degree of hearing loss, which can vary between mild to severe^12^^-13^.

The audiologic findings described in the literature show the prevalence of type A tympanometric curves, which configuration represents normality or sensorineural alteration, such as presbycusis, for instance^14^.

Once established that the elderly Brazilian population in recent years has been growing faster than the other age ranges^15^, it is necessary to better study and investigate this population in order to provide them with better quality of life.

Thus, based on the literature investigated, this study aims at tracing the audiological profile, according to the hearing threshold and the type of tympanometric curve of elderly patients who underwent auditory evaluation in an audiology clinic.

## MATERIALS AND METHODS

The present study was carried out after being approved by the Ethics Committee of the teaching institution where it was held, under protocol #O 0107.0.213.000-07. Thus, we surveyed the data according to the charts of the hearing exams, carried out in the institution by the undergraduate students, under the guidance of the speech and hearing professor in charge, between August of 2004 and August of 2007, adding up to a total of 313 hearing assessments and 626 ears.

All the assessments were done using the following equipment: Midimate 622 audiometer and the AZ7 immittance meter device, in order to evaluate the hearing threshold and to study the type of tympanometric curve, respectively.

The minimum age found was 60 years and the maximum was 94 years, with a mean age of 69.82 years. This age was selected in order to have a better sample homogeneity, as well as a complying with the greater demand from the department where the study was carried out.

According to the classification criterion utilized^16^, the frequencies of 0.25, 0.5, 1 and 2 KHz were considered low frequencies; and 3, 4, 6 and 8 KHz were considered high frequencies, and the type and degree of hearing loss were analyzed from the mean values of each one of them. Thus, since we had 626 ears, there were a total of 1252 events, since each type of frequency (highs and lows) was analyzed separately.

For immittance measuring we used the classification criteria of the tympanometric curves^17^, which classifies type A curve as the one with pressure between +50 and -70 daPa with compliance between 0.3 and 1.3 ml; As type curve - pressure between +50 and -70 daPa with compliance below 0.3 ml; type Ad curve - pressure between +50 and -70 daPa with compliance above 1.3 ml; type C curve - pressure below -70 daPa with compliance between 0.3 and 1.3 ml; type B curve - there is no maximum compliance peak.

The present study was carried out through statistical bases which characterize its intrinsic quality: sensitivity and specificity. The findings were described by analysis of frequency and occurrence, by means of descriptive graphics and tables.

## RESULTS

In most of the individuals (a little more than 60%) there were hearing thresholds of low frequency higher than the auditory threshold in the high frequencies, characterizing a descending type of curve.

As to the type of hearing loss, the results from the statistical analysis revealed that 60.78% of the population investigated had sensorineural hearing loss; 14.70% had mixed hearing loss; 2.40% had conductive hearing loss and 22.12% of the individuals had hearing thresholds within normal standards (Table 1).

**Table 1:** Type of hearing loss presented by the elderly.

TYPE	EVENTS (frequencies)	s (sensitivity)	%
Hearing thresholds within normal standards	277	0,221	22,12
Sensorineural hearing loss	761	0,607	60,78
Conductive hearing loss	30	0,024	2,40
Mixed hearing loss	184	0,147	14,70
Total	1252	1	100

As to the degree of hearing loss, we noticed they were from mild to profound, and the most prevalent one was the moderate degree (Table 2).

**Table 2:** Degree of hearing loss according to the type of loss and mean frequency.

Type of loss		Low Frequencies				High Frequencies			Total
Mild	Moderate	Severe	Profound	Mild	Moderate	Severe	Profound		
Conductive	7	5	0	0	8	9	1	0	30
Sensorineural	127	168	8	13	75	260	89	21	761
Mixed	8	58	21	4	5	30	34	24	184
Total	142	231	29	17	88	299	124	45	975

Of the 77.88% of the elderly with hearing loss, 1.76% was unilateral and 98.24% were bilateral.

As to the audiometric configurations of sensorineural hearing losses, we can see that 61.66% of the individuals had audiologic configuration suggesting presbycusis, in other words, bilateral, symmetrical and descending, of grade varying between mild to severe. On the chart that follows, we depict the auditory thresholds within normal parameters.

On Table 3 we have the types and degrees of auditory hearing loss per ear in the low and high frequencies.

**Table 3:** Hearing loss by ear and normal standards for high and low frequencies.

Hearing loss type and frequency		Conductive		Sensorineural		Mixed	
		RE	LE	RE	LE	RE	LE
Highs							
								Profound	0	0	11	10	13	11
								Severe	1	0	43	46	16	18
								Moderate	7	2	125	135	19	11
								Mild	4	4	34	41	2	3
Lows							
								Profound	0	0	8	5	2	2
								Severe	0	0	2	6	12	9
								Moderate	3	2	79	87	33	25
								Mild	4	3	68	59	8	0

**Legend:** RE - Right Ear; LE - Left Ear.

On Table 4 we notice the immittance values found in the population investigated. The type A tympanometric curve was present in 83.22% of the ears (521); while 16.3% (102) had the remaining types of tympanometric curves (“AD, AS, B, C”) and 0.48% of the ears (03) did not undergo immittance measuring.

**Table:** **Chart.** Hearing thresholds within normal standards in the high and low frequencies.

Frequencies	Normal	
	Right Ear	Left Ear
Highs	36	28
Lows	133	100

**Table 4:** Percentage of tympanometric curve type per ear.

Tympanometric curve		Frequency	Frequency percentage
	Curve A	521	83,22
	Curve Ad	28	4,48
Type of curve	Curve As	21	3,35
	Curve B	40	6,4
	Curve C	14	2,17
Not tested		2	0,38

## DISCUSSION

The higher quality of life, the improvements in health conditions and the control of chronic and infectious diseases cause a growth in the elderly population. However, the effects of the aging process on the sensorial capacities did not change, causing diseases which stem from this aging, as is the case of the hearing loss which happens to elderly, called presbycusis, which has as characteristics: bilateral, symmetrical, descending and progressive hearing loss^1^, ^2^, ^3^, ^4^, ^5^, ^6^, ^7^, ^8^, ^9^, ^10^, ^11^, ^12^,^18^, ^19^, ^20^.

According to the bibliography found, we noticed that the percentage of sensorineural hearing loss present in elderly individuals matches those in the present study. One study^1^ describes that 260 elderly were surveyed, when 64.23% had sensorineural hearing loss. Among the 331 individuals above 60 years seen in another study14, 81% had sensorineural hearing loss. These results corroborate the findings of the present paper.

Through the protocols checked, among the elderly with hearing loss, 98.24% were bilateral, which is confirmed by the literature consulted - where there is a prevalence of hearing loss in both ears of the population studied1,13-15,18-20.

Observing the data as to the type of tympanometric curves, we noticed that 83.22% of the ears studied had type A curve. In another study^14^ the authors found 78.85% of Type A curve in the elderly, being, therefore, very close values.

In relation to the audiometric configuration, 61.66% of the individuals studied had hearing loss with characteristics of presbycusis, in other words, bilateral, from mild to severe, descending, matching findings in the literature - where we find that presbycusis is characterized by sensorineural, symmetrical, descending and bilateral hearing loss for high frequency sounds (3 to 8 KHz) followed by the speech recognition difficulties^1^,^6^, ^7^, ^8^, ^9^, ^10^;^14^, ^15^,^18^, ^19^, ^20^, ^21^.

It is important to stress that presbycusis represents the natural order of things, it is part of human life and happens in accordance with the aging process^1^, ^2^, ^3^, ^4^, ^5^, ^6^, ^7^, ^8^, ^9^, ^10^; nonetheless, many elderly individuals may live well with this loss, depending on its degree and, even in the more severe cases, there are means which enable an improvement in the quality of life of this population.

## CONCLUSION

After doing this study, with elderly seen in a teaching clinic, we noticed a greater prevalence of sensorineural hearing loss, and the degree of loss varied from mild to profound, with a greater prevalence of the moderate type. Still, the high frequencies proved to be the ones more involved when compared to the low ones, in most of the individuals, characterizing descending audiometric curves, typical of presbycusis. It is also worth stressing that the most frequently found tympanometric curve was the Type A.

Therefore, we studied the hearing profile of elderly patients in a given population, thus enabling a bird's eye view on the quality of life of the people in the age range hereby investigated, and we report on a greater prevalence of sensorineural hearing loss varying from mild to profound, with a higher prevalence of the moderate degree.
